# Simulation of nitrous oxide emissions at field scale using the SPACSYS model

**DOI:** 10.1016/j.scitotenv.2015.05.064

**Published:** 2015-10-15

**Authors:** L. Wu, R.M. Rees, D. Tarsitano, Xubo Zhang, S.K. Jones, A.P. Whitmore

**Affiliations:** a Sustainable Soils and Grassland Systems Department, Rothamsted Research, North Wyke, Okehampton EX20 2SB, UK; b Scotland's Rural College (SRUC), West Mains Road, Edinburgh EH9 3JG, UK; c Ministry of Agriculture Key Laboratory of Crop Nutrition and Fertilization, Institute of Agricultural Resources and Regional Planning, Chinese Academy of Agricultural Sciences, Beijing 100081, China; d Sustainable Soils Grassland Systems Department, Rothamsted Research, Harpenden AL5 2JQ, UK

**Keywords:** Denitrification, SPACSYS, Nitrogen cycling, Simulation model, Nitrous oxide, Grassland

## Abstract

Nitrous oxide emitted to the atmosphere via the soil processes of nitrification and denitrification plays an important role in the greenhouse gas balance of the atmosphere and is involved in the destruction of stratospheric ozone. These processes are controlled by biological, physical and chemical factors such as growth and activity of microbes, nitrogen availability, soil temperature and water availability. A comprehensive understanding of these processes embodied in an appropriate model can help develop agricultural mitigation strategies to reduce greenhouse gas emissions, and help with estimating emissions at landscape and regional scales. A detailed module to describe the denitrification and nitrification processes and nitrogenous gas emissions was incorporated into the SPACSYS model to replace an earlier module that used a simplified first-order equation to estimate denitrification and was unable to distinguish the emissions of individual nitrogenous gases. A dataset derived from a Scottish grassland experiment in silage production was used to validate soil moisture in the top 10 cm soil, cut biomass, nitrogen offtake and N_2_O emissions. The comparison between the simulated and observed data suggested that the new module can provide a good representation of these processes and improve prediction of N_2_O emissions. The model provides an opportunity to estimate gaseous N emissions under a wide range of management scenarios in agriculture, and synthesises our understanding of the interaction and regulation of the processes.

## Highlights

• Microbe-controlled denitrification and N_2_O emissions were built in SPACSYS.
• Simulated outputs agreed well with a Scottish grassland dataset.
• The simulated emission factors vary with climate, management and forms of applied N.
• SPACSYS is capable of simulating the components in C and N cycling in grassland.

## 1 Introduction

Nitrogen (N) application in agriculture serves the societal need to increase agricultural production but causes long-term and deleterious environmental impacts (Sutton et al., 2011; Vitousek et al., 1997). Nitrous oxide (N_2_O) emitted to the atmosphere through agricultural activities makes an important contribution to global greenhouse gas (GHG) emissions (Reay et al., 2012) and is also involved in the destruction of stratospheric ozone (Portmann et al., 2012). The presence of nitrate- or ammonium-N is necessary to maintain production in agricultural systems but also creates the potential to generate N_2_O, and the conditions under which this occurs need to be precisely understood if appropriate mitigation strategies are to be developed.

The microbial and chemical processes of nitrification and denitrification have been reviewed in detail in the literature (Butterbach-Bahl et al., 2013; Conrad, 1996). Nitrification is generally thought to be performed by a suite of autotrophic bacteria. However, nitrification in environments that provide unfavourable conditions, e.g. acid soils, for autotrophic nitrifying bacteria and a wide range of fungi, may result from the activity of heterotrophic microorganisms Organic N is converted directly to nitrate without passing through the exchangeable ammonium pool using organic carbon (C) as an energy source (Barraclough and Puri, 1995; Odu and Adeoye, 1970). Therefore, the nitrification process includes two pathways: autotrophic nitrification and heterotrophic nitrification. Under certain circumstances (e.g. limiting soil oxygen content, low organic C content and high N content), nitrifier denitrification associated with autotrophic nitrification is increasingly being considered as an important source of N_2_O (Kool et al., 2011; Wrage et al., 2001). Also chemo-denitrification can occur through a disproportionation reaction where one nitrogen atom in NH_4_NO_3_ is reduced and the other is oxidised to produce N_2_.

Denitrification is the microbial process whereby nitrate is used as a terminal electron acceptor as it is sequentially reduced to gaseous N compounds under anaerobic conditions (Butterbach-Bahl et al., 2011; Firestone et al., 1980). Nitrous oxide, as an intermediate product of denitrification, can be released in large quantities under low oxygen concentrations in circumstances where sufficient substrate is present (nitrate and dissolved organic C) (Wrage et al., 2001). Denitrification is generally considered an anaerobic process although it has been shown that bacteria can also reduce nitrate to nitrite under aerobic conditions (Carter et al., 1995; Garrido et al., 2002).

The development of a process-based model to simulate N_2_O emission needs to take account of the interaction between biological, physical and chemical factors that control the growth and activity of microbes, as well as the soil physical and chemical environments in which they live (Davidson, 1991). Agricultural management practices can also affect the emissions (Barnard and Leadley, 2005; Rees et al., 2013). The interactions between soil physical factors and the biological processes responsible for the production and consumption in soils of GHGs including N_2_O emission have been extensively reviewed (Ball, 2013; Smith et al., 2003, 2008) and the results of these findings need to be reflected in models that simulate greenhouse gas emissions if they are to be effective.

Autotrophic nitrification and denitrification are the most important pathways to emission. Nitrifier denitrification (Ritchie and Nicholas, 1972; Wrage et al., 2001), chemo-denitrification and heterotrophic nitrification (Anderson et al., 1993) are also known to contribute to emissions under some conditions. It has been suggested that heterotrophic nitrification is not significant in agricultural soils (de Boer and Kowalchuk, 2001) nor important as a source of NO or N_2_O (Stange and Döhling, 2005). Similarly, chemo-denitrification is thought to be less important than denitrification or autotrophic nitrification as a source of N_2_O from agricultural soils (Smith et al., 1999).

There are many field and laboratory experiments which have investigated both the processes of nitrification and denitrification simultaneously. Yet these processes are difficult to isolate under field conditions and their high spatial and temporal variability makes accurate estimates of fluxes a difficult task (Groffman et al., 2006; Skiba et al., 2013). Changes in environmental conditions can result in rapid alterations in substrate concentrations or the physical environment (Saggar et al., 2013) leading to large changes in N_2_O production over a short period. As a result, models have become essential tools for integrating our current understanding of the processes with measurements of rate-controlling processes that estimate N gaseous emissions at field scale and then scaling up losses of N to the watershed or region (Boyer et al., 2006). A number of different approaches have been used to implement denitrification in N cycling models. Parton et al. (1996) categorised these as (i) the microbial growth, (ii) the soil structure and (iii) the simplified process approach. Heinen (2006) reviewed over 50 simplified models for denitrification and concluded that a universal, simple process model for denitrification cannot be built because the response functions to soil conditions are site specific. Although simple empirical relationships for N_2_O emissions can be developed (Dobbie et al., 1999; Rees et al., 2013) they often lack the ability to make predictions outside of a narrow set of environmental conditions. For this reason many attempts have been made to develop more complex process-based models which define emissions on the basis of our known understanding of the underlying processes and their kinetics (Shah and Coulman, 1978). There are a number of models in which N_2_ and N_2_O production from nitrification and denitrification at field scale are simulated using the microbial growth approach, e.g. DAYCENT (Parton et al., 1998), DNDC (Li et al., 1992), CoupModel (Norman et al., 2008), and FASSET (Chatskikh et al., 2005). However, the complexity of the processes and the models that simulate them continues to make the simulation of N_2_O and greenhouse gas emissions a difficult task, and one which remains the focus of many scientific endeavours.

The dynamics of the processes of N_2_O production are closely linked to other processes in the soil–plant–atmosphere continuum at the field scale. The SPACSYS model is a field scale, weather-driven and process-based model that includes these processes, with simulation at a daily-time-step (Wu et al., 2007). A simulated soil profile can be divided into a user-defined number of soil layers with various thicknesses. The current version includes a plant growth and development module, an N cycling module, a C cycling module, plus a soil water module that includes a representation of water flow to field drains as well as downward movement through the soil layers, together with a heat transfer module. Daily nitrification and denitrification rates are quantified using first-order kinetics modified by physical and chemical soil conditions (ammonium and nitrate concentrations, soil temperature, moisture and pH). The objectives of this paper are to describe a detailed module based on the microbial growth approach for both nitrification and denitrification that can replace the current simple routine in the SPACSYS model, and assess the performance of the improved model on the estimation of N_2_O fluxes by validation against measurements collected from a grassland site.

## 2 Methods and materials

### 2.1 Model overview

**Fig. 1:**
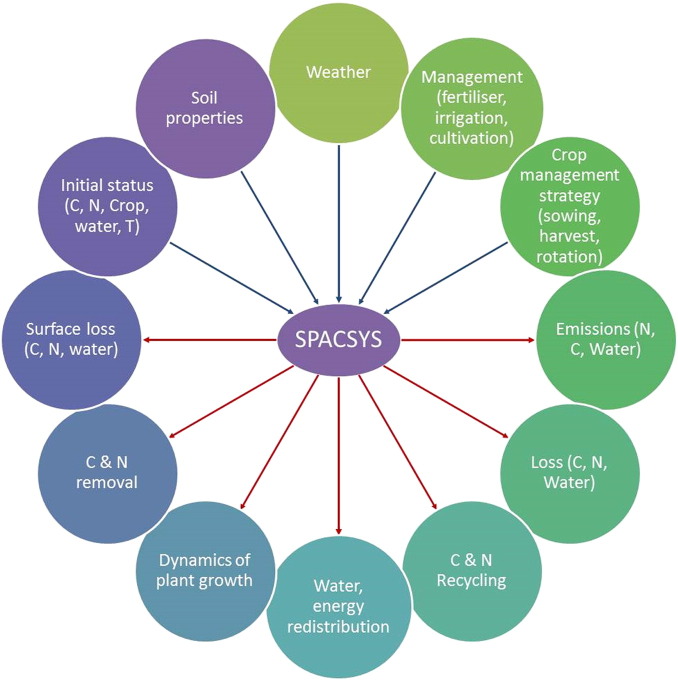
Input and output categories from the SPACSYS model.

The SPACSYS model is designed using the component object model (COM) technique and implemented in C++. All inputs (including parameters) and simulation results are organised as a database working in either the Microsoft® SQL Server 2005 or freeware MySQL environments. The major input requirement and output variables are shown in Fig. 1. The model can be applied to arable land and grassland (Bingham and Wu, 2011; Liu et al., 2013; Wu et al., 2006, 2009).

The main processes defining plant growth in the SPACSYS model are plant phenological development, assimilation, respiration, water and N uptake, partitioning of assimilation and N uptake, N fixation for legume plants, and root growth and development that can be described either with three- or one-dimensional root systems.

Nitrogen cycling coupled with C cycling in the SPACSYS model covers the transformation processes of various forms of organic matter (OM) and inorganic N. Nitrate, part of the ammonium pool and dissolved organic N are transported through the soil profile and into field drains or deep groundwater with water movement.

Adopted from the SOIL model (Johnsson and Jansson, 1991), the Richards equation for water potential and Fourier's equation for soil temperature are used to simulate water and heat fluxes. A modified Hooghoudt drainage flow equation (Hooghoudt, 1940) is used for subsurface drainage flow.

### 2.2 Estimation of nitrification and denitrification

**Fig. 2:**
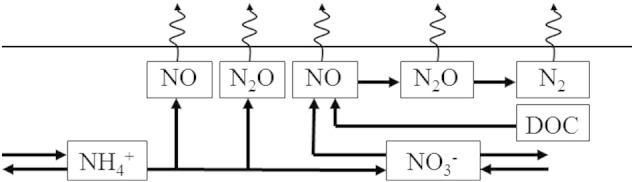
Conceptual diagram of N transformations during nitrification–denitrification processes.

Because of the uncertainties associated with minor N transformations described above, only autotrophic nitrification and heterotrophic denitrification are considered (Fig. 2). As both processes can simultaneously occur in aerobic and anaerobic microsites, respectively, substrates related each process must be allocated into two soil fractions with different aeration statuses. The concept of an “anaerobic fraction” (*f_r_*) and its implementation from the DNDC model (Li et al., 2000) was used.

#### 2.2.1 Autotrophic nitrification

Following Blagodatsky and Richter (1998), a series of equations are used to estimate microbial evolution for nitrification:

(1) $$\begin{array}{l} n_{g}=\gamma_{gn}\cdot f\left(T\right)\cdot f\left(W\right)\cdot f_{n}\left(pH\right)\cdot f_{n}\left(\mathrm{DOC}\right)\cdot f\left(NO_{3}\right)\cdot M_{b,i-1}\\ n_{d}=d_{dn}\cdot f\left(T\right)\cdot f\left(W\right)\cdot f_{n}\left(pH\right)\cdot f_{n}\left(\mathrm{DOC}\right)\cdot M_{b,i-1}\\ r_{r}=\left(\frac{1}{\varepsilon_{n}}-1.0\right)\cdot M_{b,i-1} \end{array}$$

and

(2) $$M_{b,i}=M_{b,i-1}+n_{g}-n_{d}-r_{r}$$

where *n_g_* is a gross microbial growth rate, *n_d_* is a microbial death rate, *r_r_* is a microbial maintenance respiration rate, *γ_gn_* and *d_dn_*, are the maximum gross growth rate and maximum death rate, respectively, *ε_n_* is an assimilation factor, *M*_*b*,*i*_ and *M*_*b*,*i* − 1_ are nitrifier biomass (gC m^− 2^) for the current time step and previous time step, respectively, and *f*(*T*), *f*(*W*), *f_n_*(*pH*), *f_n_*(*DOC*) and *f*(*NO*_3_) are response functions to soil temperature, water-filled pore space (WFPS), soil acidity, dissolved organic C (DOC) content and nitrate concentration, respectively.

Nitrification rate is estimated by:

(3) $$n_{n}=n_{n\max}f\left(T\right)\cdot f\left(W\right)\cdot f_{n}\left(pH\right)\cdot f\left(NH_{4}\right)\cdot M_{b,i}$$

where *n_nmax_* is the maximum rate of nitrification, and *f*(*NH*_4_) is the response function to ammonium concentration.

The effect of substrates on the autotrophic nitrification process is expressed as a Michaelis–Menten like equation:

(4) $$f\left(S_{\mathrm{con}}\right)=\frac{S_{\mathrm{con}}}{k_{m}+S_{\mathrm{con}}}$$

where *S_con_* is the substrate concentration and *k_m_* is the Michaelis constant for the substrate set to 9.45 gC m^− 3^, 16.65 and 18.53 gC m^− 3^ for DOC, nitrate and ammonium, respectively. The response functions of the process to the environmental conditions are described in detail in Section 2.2.4.

#### 2.2.2 Denitrification

Denitrifying activity is highly correlated with the content of DOC (Bremner and Shaw, 1958; Burford and Bremner, 1975; Weier et al., 1993). The reactions of the denitrification process can be described by competitive Michaelis–Menten kinetics. The rate at which each N oxide is reduced was assumed to be dependent on its concentration and a weighting factor for competition for the electron acceptors. Each reduction step involved in the denitrification process is expressed as:

(5) $$\begin{array}{l} \begin{array}{cc} d_{g,i}=\gamma_{gd,i}\cdot f_{d}\left(\mathrm{DOC}\right)\cdot f_{d,i}\left(N_{i}\right)\cdot f_{d,i}\left(pH\right)\cdot f\left(T\right)\cdot B_{d} & \left(i=1,2,3\right) \end{array}\\ d_{c,i}=\left(\frac{d_{g,i}}{Y_{nox}}+\frac{M_{Ni}\cdot N_{i}}{N_{\mathrm{total}}}\right)\cdot f\left(T\right)\cdot f_{d,i}\left(pH\right)\cdot B_{d} \end{array}$$

where *d*_*g*,*i*_ is the production rate of the *i*th denitrifier (kgC m^− 3^ d^− 1^), *d*_*c*,*i*_ is the consumption rate (kgN m^− 3^ d^− 1^) of the *i*th N oxide, *γ*_*gd*,*i*_ is a maximum growth rate (d^− 1^) of the *i*th denitrifier, *f*_*d*,*i*_(*DOC*) and *f*_*d*,*i*_(*N_i_*) are the response functions to DOC content and the concentration of the *i*th N oxide, respectively, *M_Ni_* is the maintenance coefficient on the *i*th N oxides, *N_i_* is the concentration of either NO_3_^−^, or NO or N_2_O, *N_total_* is the concentration of all N oxides, *Y_Ni_* is the maximum growth yield of the *i*th N oxides, and *B_d_* is denitrifier biomass. The response functions of the process to soil temperature and pH value are described in detail in Section 2.2.4.

The growth rate (*d_dg_*) and death rate (*d_dd_*) of total denitrifiers and the C maintenance respiration rate (*r_c_*) are estimated by:

(6a) $$d_{\mathrm{dg}}=\sum_{i=1}^{3}d_{g,i}$$

(6b) $$d_{dd}=M_{c}\cdot Y_{c}\cdot B_{d}$$

(6c) $$r_{c}=\frac{d_{\mathrm{dg}}}{Y_{c}}+M_{c}\cdot B_{d}$$

where *M_c_* is the maintenance coefficient on C and set to 0.0031 d^− 1^, and *Y_c_* is the maximum growth yield on DOC and set to 0.503 gC g^− 1^ N (van Verseveld et al., 1977).

#### 2.2.3 Gaseous emissions

The fluxes of the N gases from soils result from the balance between production and consumption processes and are highly sensitive to soil physical, chemical and biological factors (Bollmann and Conrad, 1998; Burford and Bremner, 1975; Murray et al., 2004). The stoichiometry of the emitted gas mixture (NO, N_2_O and N_2_) depends on the relative activities of the three enzymes, NO_2_^−^, NO^−^, and N_2_O-reductases (Bakken and Dörsch, 2007) which in turn are influenced by prevailing soil conditions. The emission rates were estimated following the methodology presented by Li et al. (1992).

#### 2.2.4 Response functions

A Q_10_ equation is used to quantify the effect of soil temperature on various processes with different Q_10_ values, which is consistent with temperature response functions to other processes involved in the SPACSYS model.

WFPS is estimated as (Franzluebbers, 1999; Linn and Doran, 1984):

(7) $$\mathrm{WFPS}=\frac{\theta }{1-\frac{\rho_{d}}{\rho_{s}}}$$

where *ρ_d_* is the soil bulk density (g cm^− 3^), *ρ_s_* is the particle density, typically around 2.65 g cm^− 3^, and *θ* is the volumetric water content (%).

Nitrification response to soil moisture is expressed as a quadratic function:

(8) $$f\left(W\right)=\left\{\begin{array}{cc} \min\left\{\left(1.0,(-11.25{\mathrm{WFPS}}^{2}+11.75\mathrm{WFPS}-1.9\right)\right\}\; & 0.3\leq \mathrm{WFPS}\leq 0.75\\ 0.6 & \mathrm{WFPS}<0.3\;\mathrm{or}\;\mathrm{WFPS}>0.75\text{.} \end{array}\right)$$

**Fig. 3:**
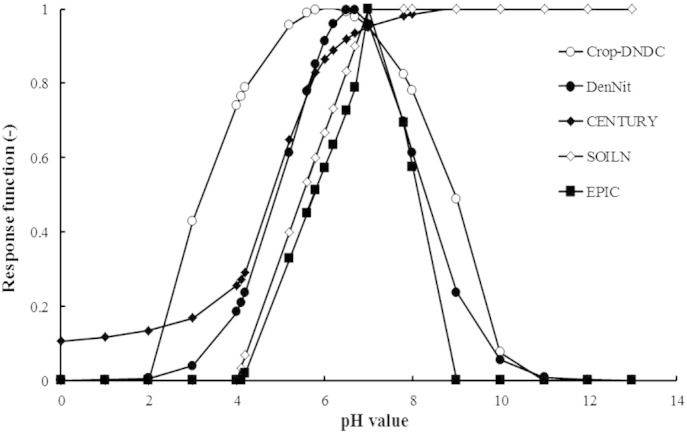
Comparison of pH response functions for the nitrification process in selected models.

There are different expressions representing the response function of nitrifying process to soil pH in simulation models (Eckersten et al., 1998; Parton et al., 1996; Reth et al., 2005; Williams, 1995; Zhang et al., 2002). It is generally understood that nitrification is detectable in soils with a pH greater than 4 (de Boer and Kowalchuk, 2001; Prosser, 1990) and the optimum pH values range from 6.6 to 7.5. If the pH values fall within this range, the response functions from various models have a similar trend (Fig. 3). Comparing datasets in the literature (Mørkved et al., 2007; Stevens et al., 1998), we found that the best simulation of the sampled data could be achieved with the exponential expression used in the DenNit model (Reth et al., 2005) and therefore have adopted that function into SPACSYS.

**Fig. 4:**
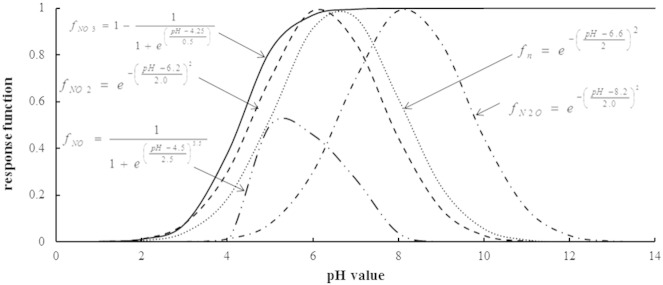
Response functions of nitrification (*f_N_*) and denitrification processes (*f_NO_*__3__: for nitrate denitrifiers; *f_NO_*: for NO denitrifiers; *f_NO_*__2__: for NO_2_^−^ denitrifiers and *f_N_*__2__*_O_*: for N_2_O denitrifiers) to soil pH.

The relationship between soil pH and denitrification is particularly complicated because pH has a varying influence on different reduction steps. Although much effort has been made to identify these relationships, published results differ and more work is needed to separate the specific effect of pH on each of the reduction step (Šimek and Cooper, 2002). Some reports found a higher rate of anaerobic NO production in an alkaline agricultural or meadow soil compared to an acid forest soil (Baumgärtner and Conrad, 1992; Remde and Conrad, 1991), whilst others reported the reverse (Krämer and Conrad, 1991). Published datasets (Murray and Knowles, 2001; Yamulki et al., 1997) were used to quantify the impact of pH on NO denitrifier growth rate. The pH response functions of NO_2_^−^ and N_2_O denitrifiers are built from another dataset (Ellis et al., 1998) whilst the impact of pH on NO_3_^−^-denitrifier growth rate follows the DNDC model (Li et al., 2000). All the response functions to pH are shown in Fig. 4.

### 2.3 Experimental site and treatments

A field experiment was carried out on a grassland soil under silage production at the Bush Estate 15 km South of Edinburgh in Scotland between 1998 and 2003. Comprehensive site details and a detailed description of the experimental design are described by Ball et al. (2004) and Jones et al. (2007). The soil type was an imperfectly drained clay loam classified as a gleysol by the FAO. Soil properties at the site, determined by McGechan et al. (1997), are shown in Table 1. The average annual rainfall was 849 mm and the mean daily temperature was 13.3 °C in July and 3.8 °C in January. In 2000 the total annual rainfall was about 1200 mm, whilst in 2003 it was only 680 mm. In general, over the experimental period, wetter conditions occurred in 1998, 1999 and 2002 whilst it was drier in 2003. The treatments considered here were: (1) zero N control (Control), (2) chemical N fertiliser (ammonium nitrate, AN), and (3) cattle slurry (Slurry). The treatments were applied in 1998 to 2000 and in 2002 and 2003 (plots were untreated in 2001, i.e. neither biomass cutting nor fertiliser application took place). The rates and timings of manure applications to the different treatment plots are given in Table 2. The site was sown with perennial ryegrass (*Lolium perenne*). Control plots contained about 50% clover (*Trifolium repens*) on an area basis, whilst fertilised plots did not contain any clover. The dates when the sward was harvested as silage were shown in Table 3.

### 2.4 Measurements for model validation

Nitrous oxide flux measurements were made using manual closed static chambers located randomly within each of the plots using the method described by Ball et al. (1997). A sampling period of approximately 60 min was used, and samples were collected in portable evacuated aluminium tubes. Gas samples were analysed by electron capture and flame ionisation gas chromatography. At each gas sampling occasion the soil water content at 6 cm depth was measured using a TDR probe (Delta-T Devices, Cambridge, England). Soil samples were collected randomly from within the plots to a depth of 0.2 m periodically throughout the experimental period.

Grass yield was measured from cuts of 8–14 m^2^ in 1999 and 2000 and 15–19 m^2^ thereafter. Plant N was determined on samples that had been dried at 60 °C and ball-milled to produce a fine flour, using a Carbo-Erba/400 automated C and N analyser.

### 2.5 Parameterisation

A soil profile was divided into 16 layers, thickness for each of the top 8 layers was 0.025 m, and then 0.15, 0.45, 0.35 and 0.2 m in order for the rest of the layers. Soil properties for each soil layer were determined by linear interpolation of the data shown in Table 1 when the model was run. Initial soil C and N pools were interpreted from Lewis et al. (2003) (Table 4). Simulations were run for a year prior to the start of the experimental period to reduce the effect of errors in the assumed initial contents of soil C, N and water. Historical daily weather data recorded over the simulation period at a nearby site were used. The distance between the met station and experimental site is less than 3 km.

SPACSYS has been previously parameterised for soil water C and N cycling processes (Wu and Shepherd, 2011). The new denitrification algorithm is characterised by several parameters, which have been determined through optimization based on root mean square error between observed data and simulation output, using data for N_2_O emissions between 1999 and 2000 with the NPK treatment. The algorithm implemented was the Multi-Objective Shuffled Complex Evolution Metropolis (MOSCEM-UA, Vrugt et al., 2003). Optimized parameters are shown in Table 5.

The model was run twice with same settings for each treatment: one with the new method of nitrification and denitrification estimation (new version thereafter) and the other with the old method (old version thereafter), to investigate if the replacement of the estimation would cause the changes of other output variables.

### 2.6 Statistical analysis

The set of statistical methods suggested by Smith et al. (1997) was used to evaluate the improvements to the denitrification and nitrification routines in SPACSYS. Seven elements in the set are included: correlation coefficient (R), root mean square error (RMSE), modelling efficiency (EF), the coefficient of determination (CD), relative error (RE), mean deviation (MD) and maximum error (ME). When an RMSE value is less than the value at the 95% confidence level, it indicates that the simulated values fall within the 95% confidence interval of the measurements. An RE value greater than the value at the 95% confidence level indicates that the bias in the simulation is greater than the 95% confidence interval of the measurement. Each of these elements provides partial insight into model performance. An appropriate evaluation of model performance is achieved by balancing different aspects of the statistical components in the set (Post et al., 2007).

## 3 Results

### 3.1 Dynamics of soil moisture

**Fig. 5:**
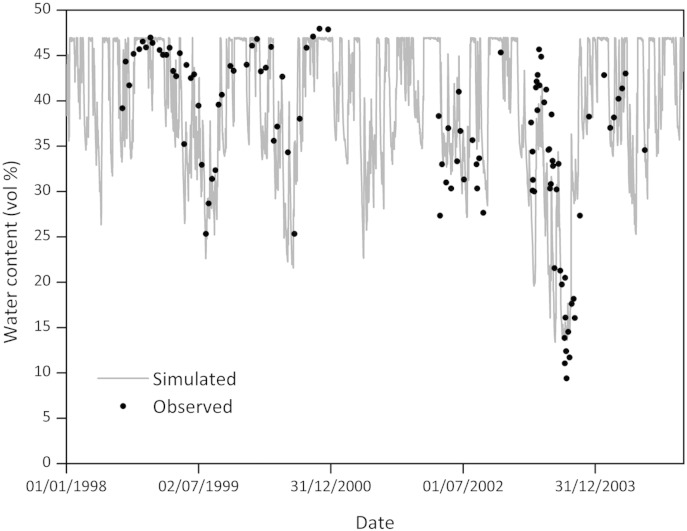
Comparison of measured (solid circle) and simulated (solid line) soil moisture at the top 10 cm soil depth under the AN treatment over the experimental period.

Soil wetness is a major driver of N transformations in the soil and an important factor for plant growth. Therefore it is essential that the model is able to simulate accurately measured soil moisture. A comparison between measured and simulated soil moisture in the top 10 cm under the AN treatment over the experimental period shows that the model was effective at simulating soil moisture across a wide range of conditions, correctly identifying baselines and extreme events (Fig. 5). The trends of soil moisture for other treatments were similar (not shown). The dynamics of soil moisture in each soil layer was almost the same with or without the new algorithm (R = 1.00) for all the treatments. The correlation coefficient indicated that the new version of the model did not change vertical water redistribution.

However, there were periods when measured and modelled values of water content were not synchronised. This was observed in July, 2003 when modelled values were significantly lower than the measurement. This fluctuation was somewhat unusual, as rainfall for this period did not support such a dramatic change. In addition, the soil moisture measurements from an adjacent plot, with similar soil characteristics and management, did not show a similar trend during the same period. Therefore, the difference could possibly be explained by measurement errors. In spite of this, the statistical analysis of soil moisture still confirmed that simulation values follow the same pattern as measured values, and fall within the 95% confidence interval of the measurements and describe the trend better than the mean of the observations (Table 6).

### 3.2 Cutting biomass and nitrogen offtake

**Fig. 6:**
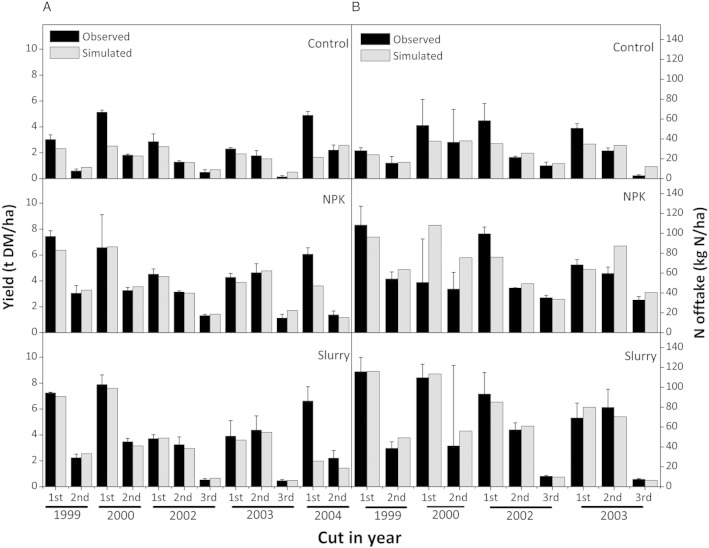
Comparison between measured and simulated dry matter (panel A) and nitrogen offtake (panel B) from all treatments (vertical lines in the graph represent standard error for measured data). No data on nitrogen offtake in 2004 available.

The simulations of dry matter and N offtake generally agreed well with measured data (Fig. 6 and Table 7). Nevertheless the model strongly under-estimated (between 40 and 70%) dry matter removal from the first cut in 2004 from all the treatments. Simulated values of dry matter and N offtake from both runs are almost identical (R = 0.99), which demonstrated that the new algorithm did not change the dynamics of biomass accumulation and N uptake.

N offtake by grasses plays an important role in soil N cycling both in terms of its magnitude and its ability to drive other processes within the N cycle. Although there was no external N input except atmospheric deposition to the plots of the Control treatment, N offtake was not much lower than the fertiliser treatments, highlighting the importance of N turnover within the soil. It should also be noted that the Control treatment included a mixture of grass and clover, providing input of biologically fixed-N. Clover can therefore increase sward N offtake as a whole. Moreover, it can also transfer its fixed N to grass through rhizodeposition and the decomposition of dead root materials, which relieves N stress for grass growth. In general, the model over-estimated N offtake for the AN and Slurry treatments.

### 3.3 Dynamics of N_2_O emissions

**Fig. 7:**
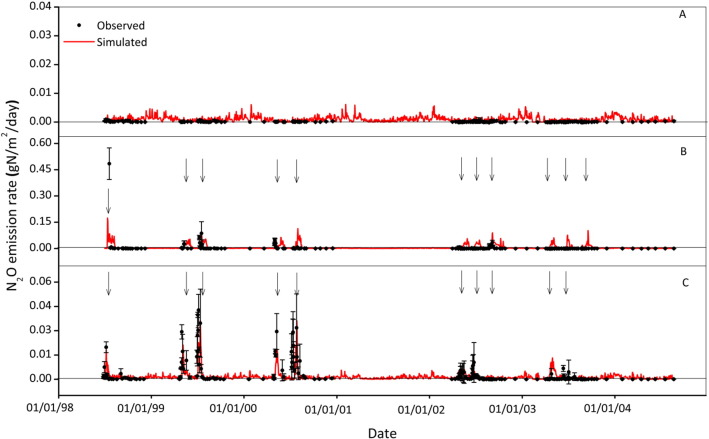
Comparison of measured (solid circle) and simulated (solid line) N_2_O emissions under the Control (panel A), AN (panel B) and Slurry (panel C) over the experimental period. The arrows indicate the dates that fertiliser or slurry was applied.

The dynamics of N_2_O emission for the various treatments demonstrated that in all the treatments, the total error in the simulated values was significantly less than the error inherent in the measured values (Fig. 7). Statistical analysis suggested that the simulations fit measured data reasonably well (Table 8) although there is no significant correlation in the Control treatment. Simulation values follow the same pattern as measured values (significant association) and describe the trend in the measured data better than the mean of the observations (positive value for EF and CD > 1) for the AN and Slurry treatments but not for the Control. Furthermore, RE values were within the 95% confidence interval of the data, indicating no bias. However, another indicator of model bias, MD, showed a slight bias towards over-estimation for the AN and Control treatments and towards under-estimation for the Slurry treatment. As N_2_O emissions were not simulated in the old version of the model, it is impossible to compare simulation results produced from the two versions.

The model was able to simulate the majority of emission peaks for the AN and Slurry treatments accurately, whereas the model is not able to explain variances in the emission data for the Control treatment. However, the model predicts N_2_O emission, for the AN treatment, shortly after the fertiliser applications in early August, 2000 and after a long dry period in mid-July and late-September, 2003, whilst the measurements do not show such a trend.

## 4 Discussion

There are many strategies available for reducing N losses from agricultural systems, but it would be difficult to verify each of them by experimentation. Simulation models can help to overcome this difficulty, and make an integrated assessment of trade-offs and contaminant swapping when comparing different management systems. The earlier simple module for estimating the total denitrification rate that was implemented in SPACSYS was unable to distinguish between the three N gases (N_2_O, NO and N_2_) due to its mathematical formulation that was largely dependent on a single coefficient to calculate emissions (Wu and Shepherd, 2011). Therefore, it is hard to have measured data to validate the rate. Although this was a valuable approach, e.g. to investigate yield response to climatic conditions, it would be a limitation for detailed N budget studies. The inclusion of the new detailed microbial-based description of processes now in the model allows an investigation of gaseous N emissions in addition to the analysis of N losses by leaching and N recycling in agricultural systems. The simulation results suggest that it may be possible to mitigate gaseous emissions by changing agricultural practices, i.e. the form, amount and timing of fertiliser N.

In GHG inventories, the amount of N_2_O-N emitted as a percentage of fertiliser N applied is defined as an emission factor (EF) by the Intergovernmental Panel on Climate Change (IPCC) and set at 1% (IPCC, 2006). Dobbie and Smith (2003b) calculated EFs that ranged from 0.4 to 6.5% at a series of experimental sites in the Great Britain and showed that annual EFs vary greatly from year to year, even with similar management practices. Published experimental data demonstrated that different forms of fertiliser N applied affect EF values, e.g. average EFs for AN-treated grassland in Scotland were 2.75 ± 0.56% but 2.12 ± 0.44% for urea treated (Dobbie and Smith, 2003a). Our simulation results showed that EFs range between 8.4 and 11% for the AN treatment but only 0.95 and 1.63% for the Slurry treatment, which is consistent with the emission factors that are controlled by climate and management (Flechard et al., 2007).

The improved model reproduced the observation of N_2_O emissions measured in the experiment with various agricultural management treatments (Fig. 7) reasonably well, as indicated by the correlation coefficients in Table 8. The trend in the fit of our simulations with measured data was similar to those using process-based models containing a similar algorithm for estimating nitrification and denitrification to the one we have implemented and reported here (e.g. Fitton et al., 2014; Ludwig et al., 2011). However, some peaks were observed in the data which were missing in the simulation results. Three potential reasons might cause this: input errors, experimental error and modelling uncertainties. Uncertain inputs of soil physical and chemical properties might contribute to the errors in estimating N_2_O emission (Nol et al., 2010). Soil properties and the sizes of state variables (pools) in a profile are essential for a simulation. Some soil parameters, e.g. saturated hydraulic conductivity and macro-pore volume, had to be estimated because measurements were unavailable. The estimated soil properties could be different from those of the experimental sites, which would inevitably have led to inaccurate estimates of N_2_O emission. Furthermore in some circumstances, measurements of driving variables for the processes and the emissions, such as water content and the concentrations of mineral N forms, were made close to, but not at the exact same location as the N_2_O flux measurements. Errors in simulating soil water redistribution in the soil (Fig. 5) are especially important. For this reason there may be discrepancies between the driving variables and the observed fluxes that the model could not be expected to identify. Thus the model represents average field conditions within the experiment but measurements incorporate spatial heterogeneity. Observations from discrete sampling points may not always represent the true mean values. The pools need initial values for a simulation run. However, the pools related to soil C and N cycles are arbitrarily determined in the model, which could result in inappropriate initial values (Wu and Shepherd, 2011). In many field studies, N_2_O emissions have demonstrated that the emissions were heterogeneous in space and time (Ambus and Christensen, 1994; Bouwman, 1996; Mathieu et al., 2006; Rees et al., 2013; Yanai et al., 2003) and, as a consequence, the pattern of N_2_O flux can be difficult to predict without a good knowledge of the spatial and temporal variability in underlying control variables. Quantifying the denitrification process is still associated with considerable levels of uncertainty, which makes its simulation more challenging (Barnard and Leadley, 2005; Firestone et al., 1980; Jetten, 2008). Denitrification would be particularly sensitive to any errors in simulating soil water distribution (Fig. 5). In addition, although there are some important environmental factors that affect the process, e.g. soil acidity, soil water content in the soil profile and soil temperature, there may be some other factors or processes, e.g. changing soil acidity after fertiliser application, and adequate representation of soil microbial processes that are not adequately represented in the model. A simulation model is always a simplification of the reality, but a successful model would be capable of capturing the general dynamics and overall effect of observed data. Inevitably, therefore there are discrepancies in the detailed description of simulated result and measurements.

## 5 Conclusions

The new module can provide a good representation of nitrification and denitrification processes and improve prediction of N_2_O emissions. Although N_2_O plays an important role in contributing to agricultural greenhouse gas emissions, the amount of emitted N from the soil–plant–atmospheric continuum only accounts for a small proportion of N cycling. Any variation in other processes might cause dramatic changes in N_2_O fluxes, e.g. plant N uptake dynamics and external N perturbation. Meanwhile, soil physical environmental changes (soil moisture and temperature) that can be driven by the status of canopy closure, plant evapotranspiration and field management also control microbial activities. A systems approach is therefore critical to the investigation of N cycling where N_2_O emissions are being characterised. It would be impossible to quantify the dynamics of the emissions over a period of years whilst excluding the interactions between the components in the continuum and other related processes. Ideally, any process-based model that simulates C and N cycling in the continuum should be fully validated on major transformation fluxes quantified in the model using a single dataset. Fully comprehensive datasets describe in all aspects of agricultural systems are probably unlikely to ever be available and the validation of complex models such as SPACSYS, is therefore, compromised by “imperfect” datasets.

## Figures and Tables

**Table 1:** Soil physical properties at the experimental site.

Depth from the top (m)	Unit	0–0.1	0.1–0.3	0.3–0.65	0.65–0.95	0.95–2.0
Dry soil bulk density	g cm^− 3^	1.53	1.53	1.52	1.52	1.52
Residual water content	%	4.1	4.1	4.1	4.1	4.1
Saturated water content	%	47	45	43	36	30
Water content at wilting point	%	12	12	12	10	10
Saturated matrix conductivity	mm d^− 1^	250	200	150	60	0.03
Saturated total conductivity	mm d^− 1^	800	500	200	3	0.36
Macro-pore volume	%	4	4	4	4	4
Pore size distribution index	–	0.19	0.19	0.18	0.16	0.14
Air entry pressure	cm	2.2	2.5	2.2	3.07	3.5
pH value		4.6	4.6	5	5	5

**Table 2:** Nitrogen application rate for the cattle slurry and chemical fertiliser treatments.

	Application date	Dry matter (%)	Total N (kg ha^− 1^)	Ammoniacal (N kg ha^− 1^)	Available (N kg ha^− 1^)
Cattle slurry	04/07/1998	5.9	220	100	122
	26/04/1999	10.0	430	154	160
	04/07/1999	3.7	190	68	110
	04/05/2000	4.2	240	113	130
	15/07/2000	4.0	200	105	110
	26/04/2002	9.2	300	127	Nd
	19/06/2002	7.2^a^	170^a^	78	Nd
	15/04/2003	7.2^a^	380	181	Nd
	18/06/2003	7.2^a^	150	62	Nd
Chemical fertiliser	04/07/1998		120	60	120
	26/04/1999		120	60	120
	04/07/1999		120	60	120
	04/05/2000		120	60	120
	15/07/2000		120	60	120
	26/04/2002		100	50	100
	19/06/2002		100	50	100
	27/08/2002		100	50	100
	15/04/2003		100	50	100
	18/06/2003		100	50	100
	15/08/2003		100	50	100

Nd = not determined.

a Estimated value based upon mean of previous applications.

**Table 3:** Sward harvest dates during 1998–2004 (same day for all treatments).

Year	1st cut	2nd cut	3rd cut
1998	21/08	–	–
1999	23/06	30/08	–
2000	29/06	13/09	–
2002	10/06	21/08	24/10
2003	04/06	20/08	23/10
2004	15/06	10/11	–

**Table 4:** Initial values of soil carbon and nitrogen pools.

Soil depth (m)	Carbon (gC m^− 2^)					Nitrogen (gN m^− 2^)				
	Humus	DOC	Microbe	Litter	Nitrate	Ammonium	Humus	DON	Microbe	Litter
0.000–0.025	938	1.92	19.16	0	0.8	0.36	69.8	0.14	0.04	0
0.025–0.050	938	1.92	19.16	7	0.8	0.36	69.8	0.14	0.04	0.68
0.050–0.075	938	1.92	19.16	10.5	0.8	0.36	69.8	0.14	0.04	1.04
0.075–0.100	938	1.92	19.16	14	0.8	0.36	69.8	0.14	0.04	1.4
0.001–0.125	822	1.92	19.16	14	0.8	0.36	74	0.14	0.04	1.4
0.125–0.150	822	1.92	19.16	20	0.6	0.28	74	0.14	0.04	2
0.150–0.175	822	1.92	19.16	12	0.4	0.2	74	0.14	0.04	1.2
0.175–0.200	822	1.92	19.16	4	0.4	0.2	74	0.14	0.04	0.4
0.200–0.350	200	0	0.4	0.5	0.04	0.04	20	0	0.002	0

**Table 5:** Optimized parameters for nitrification and denitrification in the SPACSYS model.

Parameter	Unit	Value	Equation
Maximum nitrifier gross growth rate (*γ_gn_*)	d^− 1^	4.87	1
Maximum nitrifier death rate (*d_dn_*)	d^− 1^	1.44	1
Assimilation factor (*ε_n_*)	–	0.67	1
Maximum nitrification rate of nitrifiers (*n_nmax_*)	d^− 1^	0.004	3
Maximum growth rate of NO_3_^+^ denitrifier (*γ_gd_*)	d^− 1^	13.65	5
Maximum growth rate of NO_2_^−^ denitrifier	d^− 1^	7.83	5
Maximum growth rate of NO denitrifier	d^− 1^	8.28	5
Maximum growth rate of N_2_O denitrifier	d^− 1^	8.81	5
Maximum growth yield on nitrate (*Y_Ni_*)	gC g^− 1^ N	0.65	5
Maximum growth yield on NO_2_^−^	gC g^− 1^ N	0.17	5
Maximum growth yield on NO	gC g^− 1^ N	0.75	5
Maximum growth yield on N_2_O	gC g^− 1^ N	0.24	5
Maintenance coefficient on nitrate (*M_Ni_*)	gC g^− 1^ N d^− 1^	2.16	5
Maintenance coefficient on NO_2_^−^	gC g^− 1^ N d^− 1^	8.38	5
Maintenance coefficient on N_2_O	gC g^− 1^ N d^− 1^	1.90	5
Maintenance coefficient on NO	gC g^− 1^ N d^− 1^	1.90	5

**Table 6:** Statistical analysis of model performance on dynamics of soil moisture for different treatments.

Criteria	Control	AN	Slurry
R	0.77⁎	0.76⁎	0.78⁎
RMSE (RMSE_95%_^a^)	17 (67)	19 (47)	19 (50)
EF	0.58	0.51	0.48
CD	1.82	0.91	0.92
RE (RE_95%_^b^)	− 3.05 (43)	2.78 (37)	5.87 (37)
MD	− 1.11	0.99	2.17
ME	16.58	19.60	20.49
Number of sampling events	108		

⁎ Significant association at 5% level.

a RMSE at the 95% confidence level.

b Relative error at the 95% confidence level.

**Table 7:** Statistical analysis on dry matter removal and nitrogen offtake.

	R	RMSE (RMSE_95%_^a^)	EF	CD	RE (RE_95%_^b^)	MD	ME	Number of samples
Dry matter	0.85⁎	35 (154)	0.68	1.28	12 (124)	0.41	4.65	36
N offtake	0.81⁎	40 (269)	0.54	0.85	− 10 (202)	− 4.73	60.99	30^c^

⁎ Significant association at 5% level.

a RMSE at the 95% confidence level.

b Relative error at the 95% confidence level.

c No data available for 2004.

**Table 8:** Statistical analysis of model performance on N_2_O emission rates for different treatments.

Criteria	Control	AN	Slurry
R	0.06	0.34⁎	0.50⁎
RMSE (RMSE_95%_^a^)	494 (870)	520 (736)	177 (747)
EF	− 22.25	0.04	0.20
CD	0.08	3.20	2.28
RE (RE_95%_^b^)	− 318 (546)	− 94 (220)	32 (352)
MD	− 0.0005	− 0.006	0.001
*t* for MD (*t*_2.5%_^c^)	− 3.80 (1.97)	− 2.29 (1.97)	2.73 (1.97)
ME	0.004	0.339	0.038
Number of samples	179	162	185

⁎ Significant association at 5% level.

a RMSE at the 95% confidence level.

b Relative error at the 95% confidence level.

c Critical two-tailed 2.5% *t* value.
